# Supplementary material for: Functional Characterization of MIP_07528 of Mycobacterium indicus pranii for Tyrosine Phosphatase Activity Displays Sensitivity to Oxidative Inactivation and Plays a Role in Immunomodulation
Source: Biology (Basel). 2025 May 18;14(5):565. doi: 10.3390/biology14050565 (PMC12108596; doi:10.3390/biology14050565)
Supplement: Supplementary file 1 [file biology-14-00565-s001.zip › biology-3592803-supplementary.pdf]

Gel Images used in Figure 3:

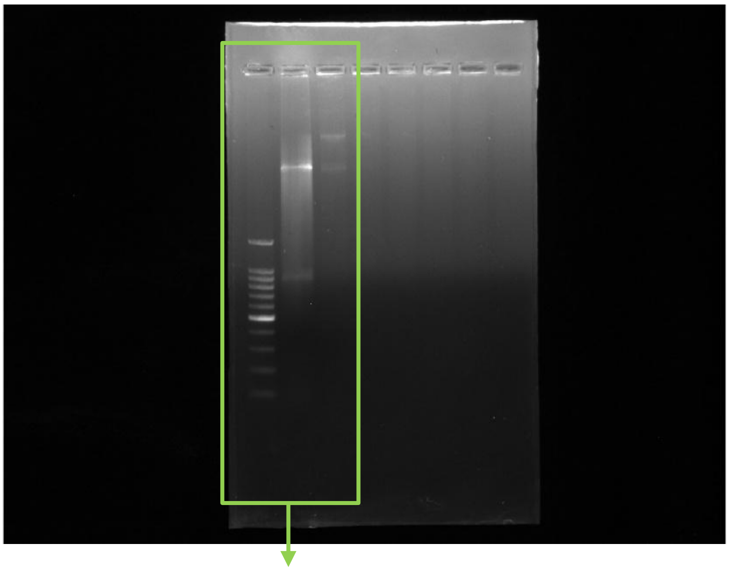

Used in Figure 3A(i):

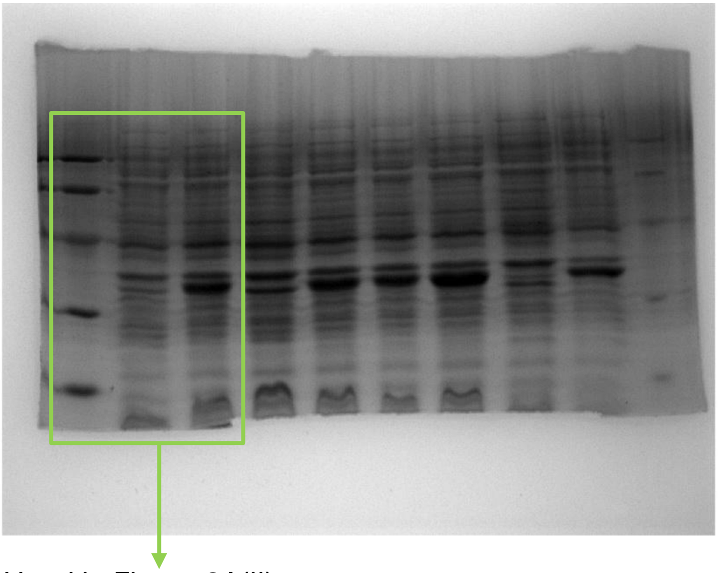

Used in Figure 3A(ii):

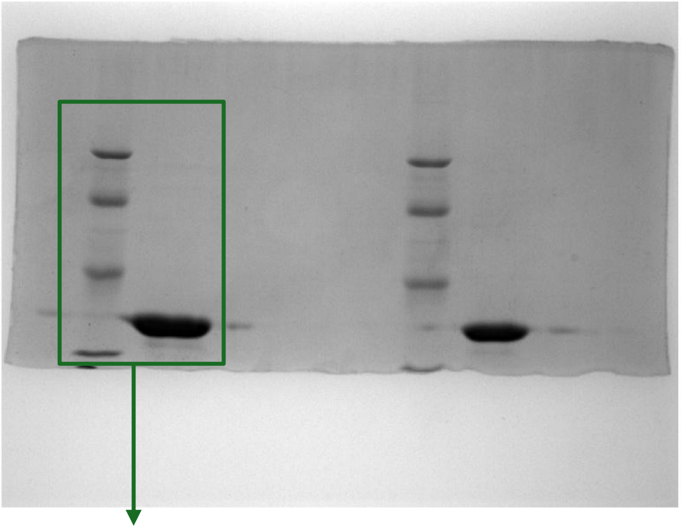

Used in Figure 3A(iii):

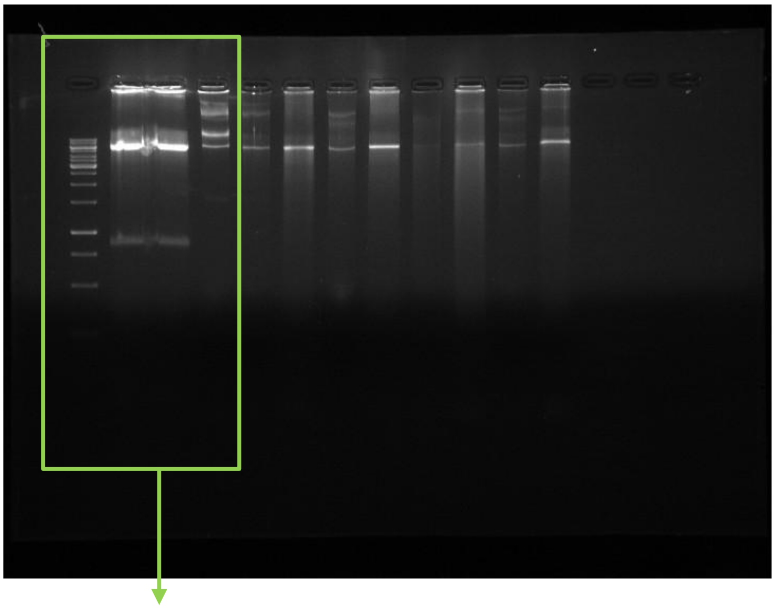

Used in Figure 3B(i):

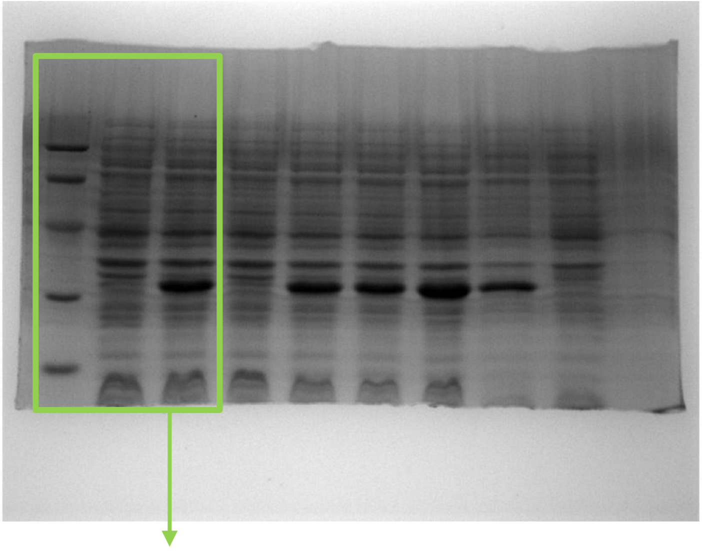

Used in Figure 3B(ii):

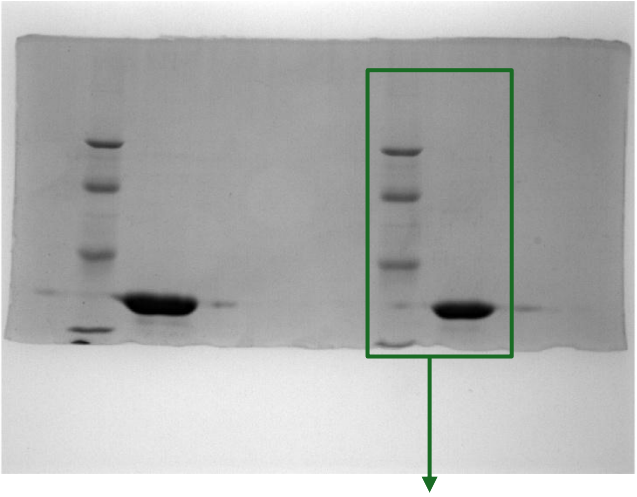

Used in Figure 3B(iii):

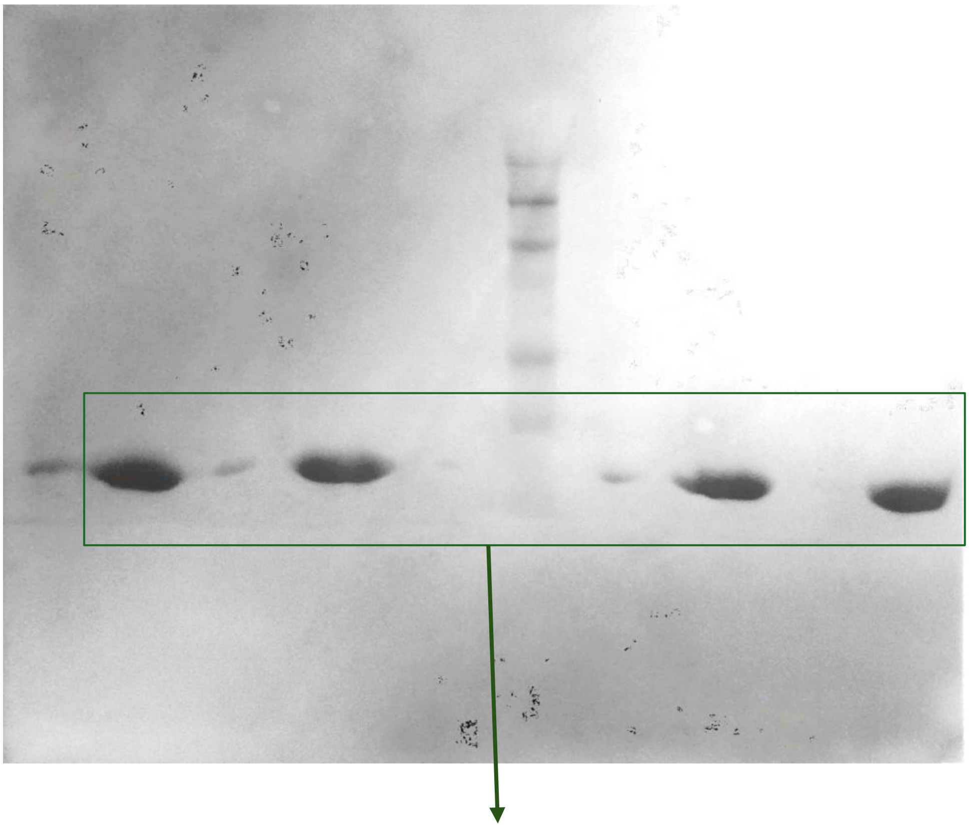

Used in Figure 3C
